# Association of Dialysis with the Risks of Cancers

**DOI:** 10.1371/journal.pone.0122856

**Published:** 2015-04-13

**Authors:** Ming Yen Lin, Mei Chuan Kuo, Chi Chih Hung, Wen Jeng Wu, Li Tzong Chen, Ming Lung Yu, Chih-Cheng Hsu, Chien-Hung Lee, Hung-Chun Chen, Shang-Jyh Hwang

**Affiliations:** 1 Division of Nephrology, Department of Internal Medicine, Kaohsiung Medical University Hospital, Kaohsiung Medical University, Kaohsiung, Taiwan; 2 Faculty of Renal Care, College of Medicine, Kaohsiung Medical University, Kaohsiung, Taiwan; 3 Instrument Technology Research Center, National Applied Research Laboratories, Hsinchu, Taiwan; 4 Graduate Institute of Medicine, College of Medicine, Kaohsiung Medical University, Kaohsiung, Taiwan; 5 Faculty of Medicine, College of Medicine, Kaohsiung Medical University, Kaohsiung, Taiwan; 6 Department of Urology, Kaohsiung Medical University Hospital, Kaohsiung Medical University, Kaohsiung, Taiwan; 7 Kaohsiung Municipal Hsiao-Kang Hospital, Kaohsiung Medical University, Kaohsiung, Taiwan; 8 Division of Gastroenterology, Department of Internal Medicine, Kaohsiung Medical University Hospital, Kaohsiung Medical University, Kaohsiung, Taiwan; 9 National Institute of Cancer Research, National Health Research Institutes, Tainan, Taiwan; 10 Division of Hepatobiliary, Department of Internal Medicine, Kaohsiung Medical University Hospital, Kaohsiung Medical University, Kaohsiung, Taiwan; 11 Institute of Population Health Sciences, National Health Research Institutes, Miaoli, Taiwan; 12 Department of Public Health, College of Health Science, Kaohsiung Medical University, Kaohsiung, Taiwan; Kaohsiung Chang Gung Memorial Hospital, TAIWAN

## Abstract

**Background:**

To increase the survival span after dialysis in patients with end-stage renal disease (ESRD), identifying specific cancer risks is crucial in the cancer screening of these patients. The aim of this study was to investigate the risks of various cancers in an incident dialysis group in comparison with a non-dialysis group.

**Method:**

We conducted a nationwide cohort study by using data from the Taiwan National Health Insurance Research Database. Patients who initially received long-term dialysis between January 1997 and December 2004, were selected and defined as the dialysis group and were matched with the non-dialysis patients (control group) according to age, sex, and index year. Competing risk analysis was used to estimate cumulative incidence and subdistribution hazard ratios (SHRs) of the first cancer occurrence.

**Results:**

After consideration for the competing risk of mortality, the dialysis group showed a significantly higher 7-year cancer incidence rate than did the control group (6.4%; 95% confidence interval [CI], 6.0%-6.7% vs 1.7%; 95% CI, 1.4%-2.1%; *P <0*.*001*).The modified Cox proportional hazard model revealed that the dialysis group had significantly association with increased risks for all cancers (SHR, 3.43; 95% CI, 3.02-3.88). The risk of cancers was dominated in younger and female patients. Specific cancer risks were significantly higher in the dialysis group particularly in the development of oral, colorectal, liver, blood, breast, renal, upper urinary tract, and bladder cancer than in the control group. Multivariable stratified analyses confirmed the association between long-term dialysis and cancer in all subgroups of patients.

**Conclusions:**

Dialysis is associated with a higher risk of cancer in patients with ESRD. However, cancer screening in ESRD population should be a selective approach, based on individual patient health condition and life expectancy.

## Introduction

The number of patients with end-stage renal disease (ESRD) worldwide has increased rapidly over the past few decades. By 2010, the estimated ESRD population was over 2 million, and the survival of these patients depends on expensive dialysis treatments and transplantation [1]. Advanced dialysis techniques increase patients’ life span; however, these techniques also increase the risk of complications. In recent years, researchers have increasingly examined specific complications of long-term dialysis, for example, the development of cancer rather than cardiovascular diseases in dialysis patients [2–10]. Studies on the risks of various cancers in dialysis patients are crucial in the development of cancer screening strategies, particularly in countries where the prevalence and incidence of ESRD is high.

Previous studies have showed the higher risks of various cancers in dialysis patients than general population, such as cancers of the liver [7, 9, 10], kidneys [2–4, 7–10], urinary tract [4, 8], bladder [2, 3, 7, 9], thyroid [2, 7, 8], and other organs [2, 8–10]. However, most the studies that reported these associations were either limited by the small scale of the study [8], inclusion of prevalent dialysis patients [10], or estimation of the risk ratio by using the excess standardized incidence ratio [2, 3, 7]. The shortcomings of these approaches were an inability to control the confounding effects on the outcome of interest and failure to obtain accurate comparison of risks for generalization to entire populations.

The purpose of this nationwide cohort study was to evaluate the association of dialysis and the risks of cancers. We used an incident dialysis population matched with the non-dialysis patients according to age, sex, and index year to explore this association.

## Materials and Methods

### Study population

We conducted a population-based cohort study through the Taiwan National Health Insurance Research Database (NHIRD), which contains claims information and health care data on more than 99% of the population of Taiwan. The accuracy of major disease diagnoses recorded in the NHIRD has been validated [11–13].This study was approved by the ethical review board of Kaohsiung Medical University Hospital (KMUH-IRB-EXEMP-20130014). All the studying processes were obeying the Declaration of Helsinki without violation any ethical principle. Because the patient identifiers in this national dataset were scrambled for research use, the review board waived the requirement for written informed consent.

We retrieved records on all patients who have received long-term dialysis since 1997 and comparable controls, who were matched with the dialysis patients according to age, sex, and index year, The patients who initially received dialysis according to the International Classification of Diseases, Ninth Revision, Clinical Modification [ICD-9-CM] codes for chronic renal failure under regular dialysis in the Registry for Catastrophic Illness Patient Database (RCIPD), a subset of the NHIRD (S1 Table). We obtained the initiation date of dialysis treatment for each outpatient by tracking the specific payment codes (S2 Table) from the NHIRD for the treatment and care received during hemodialysis and peritoneal dialysis.

The comparable controls were selected from the Longitudinal Health Insurance Data 2000 (LHID2000), which contains all claims data on 1 000 000 people. The subjects in LHID2000 were randomly sampled from the entire insured population from 1996 to 2000, and no differences in the age and sex distributions between the LHID2000 and the general population were observed. Patients (n = 996 346) who never received dialysis during 1997–2004 and had at least 1 outpatient visit (a total of 6 656 719 records) were considered potential matching controls.

A matching procedure was used to enhance the comparison between the dialysis and control groups. The index year was defined as the year of dialysis for the dialysis group, whereas the index year was the year of an outpatient visit for the control group. Age was calculated from the date of birth to the initiation date of dialysis for the dialysis group and from the date of birth to the date of the outpatient visit for the control group. Because the patients in the dialysis group were significantly older than those in the control group, we matched each patient with the control group according to age (within ± 2 y), sex, and index year by using the existing programs [14]. In each pair, the index date was the initiation date of dialysis in the dialysis group and the corresponding first outpatient visit date in the control group.

### Main outcome measurements

Cancer occurrence was determined according to the ICD-9-CM codes from the RCIPD, and the first registry date was considered the date of cancer occurrence. All cancer registries in the database contained either surgical pathological information or a typical image presentation. Details on cancers at various sites, including oral, esophageal, gastric, colorectal, liver, pancreatic, lung cancer, hematological, breast, cervical, prostate, upper urinary tract, and bladder cancers, are shown in S1 Table. Because of the lack of accuracy in determining kidney cancer on the basis of the diagnostic code (ICD-9-CM code 189.0) [15], we used the surgical procedure codes (S2 Table), including those for radical nephrectomy, laparoscopic nephrectomy, and partial nephrectomy, to confirm renal cancer. Death was defined as death during hospitalization or self-discharges from hospitalization, without any outpatient follow up thereafter.

### Covariate assessment

In covariate assessment, factors that possibly confound the risk of cancer occurrence were included. We obtained data on patient characteristics such as age, sex, index year (1997–1998, 1999–2000, 2001–2002, 2003–2004), insurance region (north, center, south, east), and urbanization (urban, suburban, rural) [16]. The Charlson comorbidity index (CCI) score was calculated for all diseases listed in a previous study [17]. Potential confounding comorbidities, which are listed using ICD-9-CM codes in S1 Table, included diabetes, hypertension, cerebrovascular disease, ischemic heart disease, and congestive heart disease. These were identified on the basis of ≥2 outpatient visits or ≥1 inpatient visit within 1 year before the index date.

### Statistical Analysis

The mortality rate was considerably higher in dialysis patients than in non-dialysis patients. Therefore, the competing risk approach was used to calculate an accurate cancer incidence rate. The death-adjusted cumulative incidence for the marginal probability of cancer was obtained, and the cumulative incidences in the competing risk data were compared using the modified log-rank test [18].

To compare the subdistribution hazard ratios (SHRs) for various sites of cancer occurrence between the dialysis and control group, multivariable analyses were conducted using modified Cox regression hazard models [19]. To represent the competing risk hazard for each event, we adjusted for age, sex, index year, insurance region, urbanization, comorbidities, and the CCI score.

To determine the association between the duration of dialysis and cancer occurrence, we estimated SHR of cancer stratifying the follow-up duration. In addition, we used multivariable stratified analyses of different subgroups to validating these findings. Age-specific and sex-specific SHRs for various sites of cancer were estimated and are reported in S1–S4 Figs.

Data management and analyses were performed using SAS 9.3 (SAS Institute Inc., Cary, North Carolina, USA) software. The cumulative incidence in competing risk analyses was calculated using the cmprsk package of R [20]. A 2-sided *P* value lower than. 05 was considered statistically significant.

## Results

### Demographic characteristics of the observed cohort

**Fig 1:**
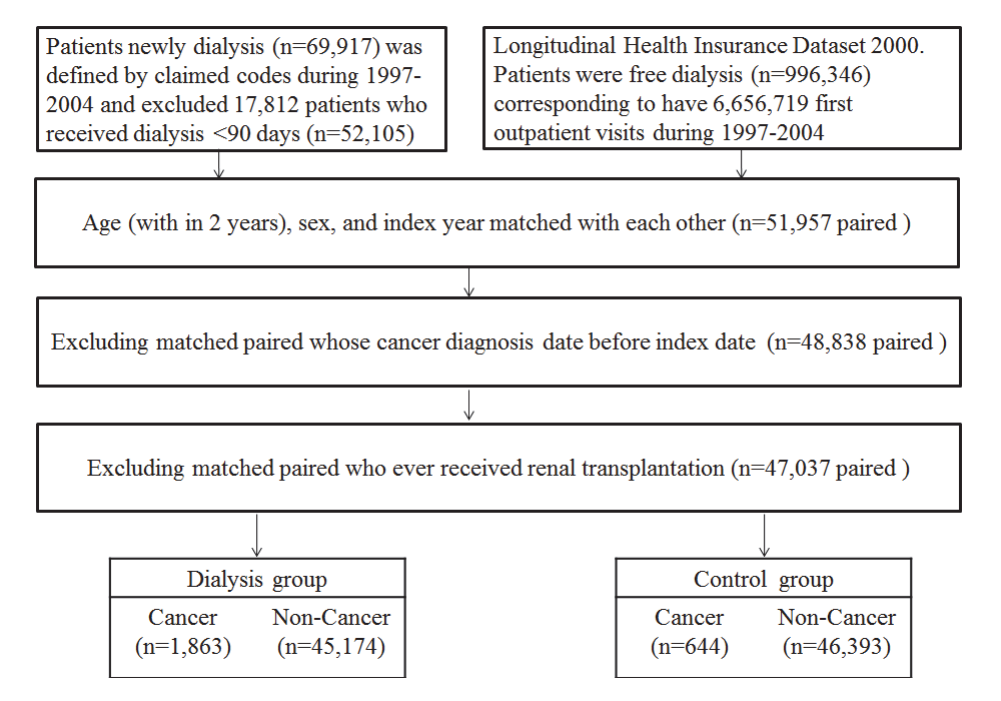
Flow diagram illustrating the selection of patients and controls.

We first selected 69 917 patients who newly received dialysis during 1997–2004. After patients who received dialysis for fewer than 3 months were excluded, 52 105 dialysis patients were included, and 51 957 (99.7%) age (±2 y)-, sex-, and index-year-matched patients were selected from the non-dialysis population as the control group. We excluded 3 119 matched pairs in which one of the patients developed cancer before the index date. Finally, 47 037 patients matched according to age, sex, and index year were included in the final analysis (Fig 1).

The demographic characteristics, comorbidities, CCI scores, and follow-up durations of the study cohort are shown in Table 1. The mean (SD) follow-up duration for the dialysis group was 3.0 (2.1) years, and the median (interquartile range [IQR]) was 2.5 (1.2–4.4) years; these durations were significantly lower than those for the control group, for which the mean (SD) was 4.2 (2.2) years and the median (IQR) was 4.0 (2.0–6.0) years. The proportions of patients who lived in the southern insurance region (34.6% vs 27.7%) and urban areas (75.6% vs 56.7%) were higher in the dialysis group than in the control group.

**Table 1:** Characteristics and prescription drugs in dialysis group and the age-, sex-, and index year-matched control group.

	Dialysis group (n = 47,037)	Control group (n = 47,037)	P-value
Age, mean (SD), y	58.2 (15.1)	57.0 (15.4)	<0.001
Sex (%)			1.00
Male	47.2	47.2	
Female	52.8	52.8	
Index year (%)			1.00
1997–1998	23.7	23.7	
1999–2000	24.6	24.6	
2001–2002	27.8	27.8	
2003–2004	23.9	23.9	
Insured region (%)			<0.001
Northern	42.1	47.4	
Central	20.7	21.4	
Southern	34.6	27.7	
Eastern	2.7	3.5	
Urbanization (%)			<0.001
Urban	75.6	56.7	
Suburban	22.2	31.8	
Rural	2.2	11.6	
Major coexisting disease (%)			
Diabetes	36.9	6.6	<0.001
Hypertension	70.5	16.5	<0.001
Cerebrovascular disease	11.2	4.0	<0.001
Ischemic heart disease	20.9	5.9	<0.001
Congestive heart failure	23.3	10.9	<0.001
Charlson score			<0.001
Mean (SD)	3.9 (2.1)	3.3 (9.0)	
Median (IQR)	3 (2–5)	0 (0–0)	

Data are expressed as percentages. Chi-Square test and Wilcoxon two-sample test is used to test the differences between the dialysis group and the control group for categorical and continuous variables. Statistical significance is defined as p value less than 0.05.

The presence of comorbidities, namely diabetes, hypertension, cerebrovascular disease, ischemic heart disease, and congestive heart failure, was significantly higher in the dialysis group than in the control group. The mean (SD) CCI score was 3.9 (2.1) for the dialysis group and 3.3 (9.0) for the control group, and the median (IQR) was 3 (2–5) for the dialysis group and 0 (0–0) for the control group.

### Seven-year cumulative incidence and risk of cancer

**Fig 2:**
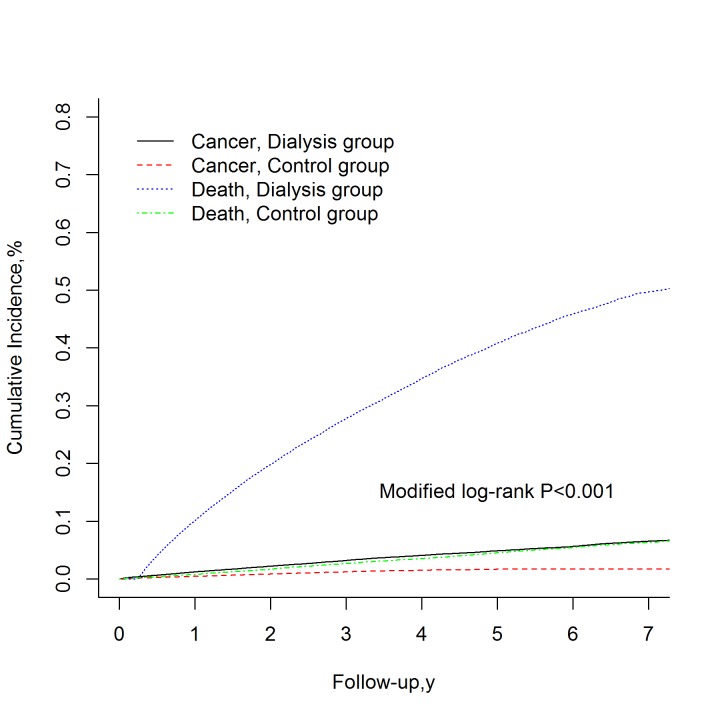
Cumulative incidence for cancer and mortality. Data were compiled after consideration for competing mortality. The cumulative rate between dialysis and control group were calculated using Modified Kaplan-Meier and Gray methods [30] and test their difference between groups by modified log-rank test. The Modified log-rank P value for comparing the cumulative incidence for cancer in dialysis and control group is less than 0.001.

During the observation period, 1863 (4.0%) patients in the dialysis group and 644 (1.4%) patients in the control group developed cancer. The incidence of bladder cancer was the highest in the dialysis group, accounting for approximately 21%, whereas the proportion of this cancer was only 3% in the control group during the follow-up years. The mean age for cancer diagnosis in the dialysis group was 5 years lower than that in the control group, and the number of female patients diagnosed with cancer was higher in the dialysis group than in the control group (S3 Table and S4 Table). Death before cancer occurrence was considered a competing risk. The cumulative incidence of cancer after adjustment for competing mortality was significantly higher in the dialysis group (cumulative incidence, 6.4%; 95% confidence interval [CI], 6.0%–6.7%) than in the control group (cumulative incidence, 1.7%; 95% CI, 1.4%–2.1%; *P* < 0.001) (Fig 2). The dialysis group had a significantly higher risk of all cancers (SHR, 3.43; 95% CI, 3.02–3.88; *P* < 0.001) than the control group did. Furthermore, these cancers particularly occurred in the oral cavity (SHR, 2.50; 95% CI, 1.43–4.39; *P* = 00.001), colon (SHR, 2.34; 95% CI, 1.62–3.39; *P* <0.001), liver (SHR, 1.88; 95% CI, 1.35–2.61; *P* <0.001), blood (SHR, 2.30; 95% CI, 1.20–4.42; *P* = .01), breast (SHR, 4.21; 95% CI, 2.51–7.06; *P* < 0.001), kidneys (SHR, 40.31; 95% CI, 13.35–121.75; *P* < 00.001), upper urinary tract (SHR, 61.33; 95% CI, 18.58–202.45; *P* <00.001), and bladder (SHR, 41.95; 95% CI, 25.10–70.11; *P* <00.001) (Table 2).The risk of developing cancer in the dialysis group was consistently higher in all follow-up periods than that in the control group. The proportion of specific cancer in patient with cancer showed a great difference between groups during the first year follow up. Cancers of urinary system (kidney, upper urinary tract, and bladder cancers) accounted for considerable proportion of cancers in dialysis group (34.1%) compared to control group (5.3%) (data not shown). The highest risk of cancer in the dialysis group was observed ≥4 years after dialysis (SHR, 15.2; 95% CI, 10.8–21.5; *P* < 0.001) (Table 3).

**Table 2:** Incident density of various sites of cancers.

Sites (ICD-9-CM codes)						
Groups	Case	I^a^	Crude HR (95% CI)	p-value	aSHR^b^ (95% CI)	p-value
Oral cancer (140–149)						
Control	35	0.17	1 [Reference]	<0.001	1 [Reference]	0.001
Dialysis	100	0.55	3.84 (2.61–5.65)		2.50 (1.43–4.39)	
Esophageal cancer (150)						
Control	14	0.07	1 [Reference]	0.1376	1 [Reference]	.42
Dialysis	17	0.09	1.72 (0.84–3.50)		0.64 (0.22–1.87)^#^	
Gastric cancer (151)						
Control	57	0.28	1 [Reference]	0.0124	1 [Reference]	.44
Dialysis	66	0.36	1.58 (1.10–2.25)		1.25 (0.71–2.2)	
Colorectal Cancer (153–154)						
Control	93	0.46	1 [Reference]	0.0124	1 [Reference]	<0.001
Dialysis	190	1.04	2.80 (2.18–3.59)		2.34 (1.62–3.39)	
Liver cancer (155)						
Control	84	0.41	1 [Reference]	<0.001	1 [Reference]	.0002
Dialysis	271	1.48	4.38 (3.42–5.60)		1.88 (1.35–2.61)	
Pancreatic cancer (157)						
Control	22	0.11	1 [Reference]	0.0724	1 [Reference]	.14
Dialysis	8	0.04	0.48 (0.21–1.07)		0.36 (0.09–1.41)	
Lung cancer (162)						
Control	85	0.42	1 [Reference]	0.2659	1 [Reference]	.90
Dialysis	75	0.41	1.19 (0.87–1.63)		0.97 (0.59–1.6)	
Blood cancer (200–208)						
Control	29	0.14	1 [Reference]	0.0003	1 [Reference]	.01
Dialysis	55	0.30	2.31 (1.47–3.62)		2.3 (1.2–4.42)	
Breast cancer (174)						
Control	40	0.20	1 [Reference]	<0.001	1 [Reference]	<0.001
Dialysis	110	0.60	3.79 (2.63–5.45)		4.21 (2.51–7.06)^#^	
Cervical cancer (180)						
Control	36	0.18	1 [Reference]	0.0007	1 [Reference]	.29
Dialysis	56	0.31	2.08 (1.37–3.17)		1.42 (0.74–2.72)^#^	
Prostate cancer (185)						
Control	23	0.11	1 [Reference]	0.1054	1 [Reference]	.36
Dialysis	28	0.15	1.58 (0.91–2.75)		0.67 (0.28–1.59)^#^	
Kidney cancer (189.0)^$^						
Control	4	0.02	1 [Reference]	<0.001	1 [Reference]	<0.001
Dialysis	87	0.48	29.08 (10.67–79.24)		40.31 (13.35–121.75)	
Upper urinary tract cancer (189.1 and 189.2)						
Control	4	0.02	1 [Reference]	<0.001	1 [Reference]	<0.001
Dialysis	107	0.58	47.52 (15.41–146.55)		61.33 (18.58–202.45)	
Bladder cancer (188)						
Control	18	0.09	1 [Reference]	<0.001	1 [Reference]	<0.001
Dialysis	388	2.12	32.26 (20.10–51.76)		41.95 (25.1–70.11)	

Time to follow up is 183,184 and 202,528 person-years in dialysis and control group.

Abbreviation: SHR, subdistribution hazard ratio; CI, confident interval; aSHR, adjusted subdistribution hazard ratio.

^a^Incident rate (per 1,000 person-year).

^b^Model was adjusted age, sex, index year, urbanization, diabetes, hypertension, cerebrovascular disease, ischemic heart disease, congestive heart failure, and Charlson score by competing risk model.

^$^Kidney cancer was identified by ICD-9 code with surgical procedures including radical nephrectomy, laparoscopic nephrectomy, and partial nephrectomy.

^#^ means that sex for the specific event was not included in the model.

**Table 3:** Cancer Occurrence in Relation to Dialysis, by Follow-up Duration.

	Control group			Dialysis Group				
Time	Case	PY	I^a^	Case	PY	I^a^	aSHR (95% CI)^b^	p
All observed period	644	202,528	3.18	1,863	183,184	10.17	3.43 (3.02–3.88)	<0.001
Follow-up duration								
<1y	226	46,245	4.89	568	42,747	13.29	1.69 (1.36–2.11)	<0.001
1–2 y	169	40,831	4.14	383	32,567	11.76	2.67 (2.04–3.48)	<0.001
2–3 y	142	33,747	4.21	319	23,784	13.41	2.63 (1.87–3.41)	<0.001
3–4 y	80	26,917	2.97	223	16,919	13.18	4.04 (2.83–5.77)	<0.001
>4y	45	49,106	0.92	372	23,979	15.51	15.2 (10.8–21.5)	<0.001

Abbreviation: PY, person-years; I, incidence; aSHR, adjusted subdistribution hazard ratio; CI, confident interval.

^a^Incident rate (per 1,000 person-year).

^b^Models were adjusted age, sex, index year, urbanization, diabetes, hypertension, cerebrovascular disease, ischemic heart disease, congestive heart failure, and Charlson score by competing risk model.

### Stratified analysis

**Fig 3:**
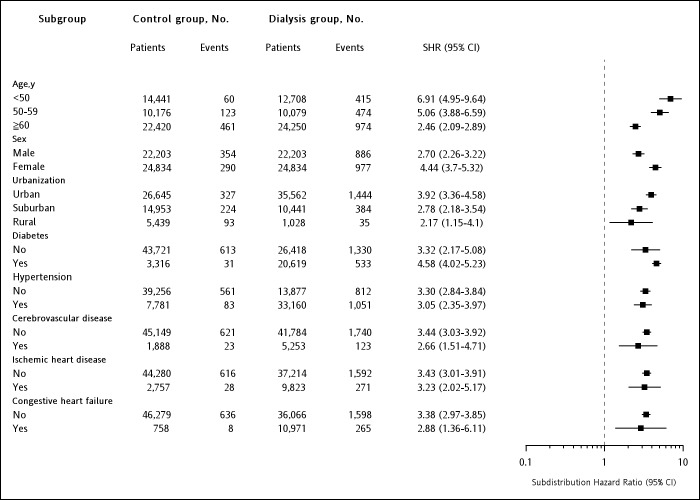
Multivariable stratified analyses for the association between dialysis and cancer. Abbreviation: SHR, subdistribution hazard ratio; CI, confident interval.

We confirmed the increased risks of all cancers in all stratified analyses (Fig 3). Higher risks were observed in patients aged <50 years (SHR, 6.91; 95% CI, 4.95–9.64; *P* < 0.001) and 50–59 (SHR, 5.06; 95% CI, 3.88–6.59; *P* < 0.001) than in those aged ≥60 years (SHR, 2.46; 95% CI, 2.09–2.89; *P* <00.001). The risks were higher in female patients (SHR, 4.44; 95% CI, 3.70–5.32; *P* <00.001) than in male patients (SHR, 2.7; 95% CI, 2.26–3.22; *P* < 00.001). The risk of overall cancer remained significantly higher in the subgroups of the dialysis group stratified according to various comorbidities, including diabetes (SHR, 4.58; 95% CI, 4.02–5.23; *P* < 0.001), hypertension (SHR, 3.05; 95% CI, 2.35–3.97; *P* < 0.001), cerebrovascular disease (SHR, 2.66; 95% CI, 1.51–4.71; *P* < 0.001), ischemic heart disease (SHR, 3.23; 95% CI, 2.02–5.17; *P* < 0.001), and congestive heart disease (SHR, 2.88; 95% CI, 1.36–6.11; *P* < 0.001).

The risks of cancers at various sites stratified according age (using 60 y as the cutoff value) and sex showed marked differences in oral and gastric cancers in patients aged <60 years than in those aged >60 years, and a significantly higher incidence of upper urinary tract and bladder cancers was observed in female patients than in male patients (S1–S4 Figs).

## Discussion

Through our large-scale nationwide cohort study and the competing risk analysis, we clearly showed that the risk of cancer in the Taiwanese incident dialysis population is increased. We also identified site-specific cancers, such as oral, colorectal, liver, breast, upper urinary tract, renal, bladder, and blood cancers, which were frequently observed in this population. Younger and female patients were at a higher risk than older and male patients, respectively.

The relationship between dialysis and the high risk of cancer has been widely investigated in several countries [2, 3]. However, an increasing risk of specific cancers in dialysis patients remains substantially inconclusive. An international collaborative study from the United States, Europe, Australia, and New Zealand reported an increased risk of cancers of the bladder, urinary tract, thyroid, and other endocrine organs in dialysis patients [3]. In Japanese dialysis patients, the excessive risk was observed on renal cancer, but not on upper urinary tract cancer [4]. Even within single country, studies that reported significant differences in specific cancer incidences in dialysis patients compared with the general population were not in complete agreement [7, 8, 10]. The discrepancy may be caused by various factors involved in the method for estimating the risk. The standard incidence ratio was often used in these studies, but this could be a problem if the ratio is not effectively controlled for confounding effects on the outcome of interest. Few studies have used an appropriate control cohort to identify the relative risk of cancer in incident dialysis patients. The traditional Cox regression model can be biased because the competing event, such as death, was significantly higher in the dialysis group than the general control group. Our study had the advantages of matched controls for age, sex, and index year; effective control of the confounding effects; and the use of the competing risk model for SHR estimation, potentially strengthening the reliability of the results.

The causes for the increased risks of specific cancers in the dialysis population have not been elucidated adequately. This could be attributed to differences in carcinogens to various cancers among countries. *Aristolochia* species of plants were either frequently mixed or misused in Southeastern Europe and Asia [21]. Plants containing aristolochic acid were considered a causative agent of renal damage and urinary tract cancer. This is a possible reason for the increased incidence of urinary tract cancer in Asian dialysis patients. The specific cancers including kidney, upper urinary tract, and bladder cancers showed high proportions in dialysis group during the first year follow up. These cancers occurred and accompanied with kidney disease even before dialysis, which implied certain causal factors linking the renal damage and tumorogenesis. The mechanisms may have a long-term period of development covering the whole process of kidney disease progression. From the similar results in current researches, young age and female are risk factors for aristolochic acid induced renal disease and cancers [22–24]. It suggests that cancer screening in patient with renal disease should be selective. The higher risk of oral, liver, colorectal, breast, and blood cancers shows in dialysis group compared to control group in the study, but the reason has not been well clarified. One large study considers viruses may play a key role on human papillomavirus-related- cancer such as vulvovaginal cancer in patient with ESRD [25]. The higher prevalence of Hepatitis C viral infections in patient with advance chronic kidney disease in Taiwan had been reported in our previous study [26]. It is likely that Hepatitis C virus may be involved in the progression and development of liver cancer in dialysis patients. Furthermore, chronic infection and inflammation, impaired functioning of the immune system, nutrition deficiency, and shortage of DNA repair mechanisms, which frequently appeared in dialysis patient [27, 28], may also contribute the tumorigenesis of these cancers.

Our results are consistent with those of related studies [2, 7, 9], which reported that the risk of cancer was greater in younger dialysis patients. One possible explanation for this is a stronger inflammatory response in young patients upon external stimulation. This response is likely to trigger tumorigenesis; however, the underlying mechanism requires further elucidation. Female dialysis patients have higher cancer risk than male patients; this observation is consistent with the early findings of other studies [8, 9]. Previous studies have reported that females are at higher risk for kidney disease and urinary tract cancer caused by Chinese herbs than are males [29–32]. These results suggest that sex, at least partly, influences the initiation of renal disease and urinary tract cancer. However, further research should be conducted to clarify the association of sex with cancerogenesis in dialysis patients. This study had several strengths. First, the competing risk for death approach substantially increased the ability to identify the risk of specific cancers. Second, the use of age-, sex-, and index year-matched control groups increased the comparability and reduced time-related bias of the observations. Third, patients in the study group had incident dialysis and were followed up for the development of incident cancer after dialysis. The time sequence between these two diseases obviously improves through this study design. Fourth, we stratified our population in subgroup analyses, and the results were similar, supporting the hypothesis that the risk of cancer in the dialysis population is increased.

Nevertheless, our study had certain limitations. First, it was impossible to confirm the cancer diagnoses according to histopathology reports in the NHIRD. However, the accuracy of diagnoses in the RCIPD can be considered valid because pathological and/or cytological evidence is required for approval. Second, we were unable to obtain major information on the patients’ history regarding smoking and other lifestyle choices. This limitation has been noted in previous observational studies that analyzed claim data, and the studies revealed a compromise between sample sizes and comprehensive collection of confounding factors. These results were obtained despite sex being assumed, in part, as a surrogate variable for smoking for adjustment in the models, as mentioned in a previous study [33]. Third, although an increase in the risk of all and site-specific cancers was observed in the dialysis population, these results must be explained in greater detail. Dialysis patients may be more aware of cancer because they are more frequently monitored for cancer development by medical staff, frequently visit the hospital for dialysis, and have easy access to cancer screening when abnormal symptoms arise. Finally, although the comparability between dialysis group and control group was substantially improved through matching their age and sex, we still cannot rule out the possibility that residual confounders could influence on our risk estimation.

## Conclusion

In summary, we proved that the risks of all cancers and site-specific cancers (i.e., oral, colorectal, liver, breast, upper urinary tract, kidneys, bladder, and blood cancers) are increased in the incident dialysis population. Younger age and female sex increase susceptible to cancers. These findings suggest that cancer screening in ESRD population should be a selective approach, based on individual patient health condition and life expectancy.

## Supporting Information

S1 Fig The association between dialysis and various sites of cancers in aged younger than 60 years. (TIF)

S2 Fig The association between dialysis and various sites of cancers in aged equal and older than 60 years. (TIF)

S3 Fig The association between dialysis and various sites of cancers in male. (TIF)

S4 Fig The association between dialysis and various sites of cancers in female. (TIF)

S1 Table The corresponding ICD-9-CM codes for the diagnosis of disease in the study. (DOCX)

S2 Table The payment codes for the clinical treatment providing by Taiwan National Health Insurance. (DOCX)

S3 Table Diagnosed age and proportion of female in dialysis patient with occurring cancer. (DOCX)

S4 Table Diagnosed age by sex in dialysis patient with occurring cancer. (DOCX)
