# Supplementary figures and images for: Association of Dialysis with the Risks of Cancers
Source: PLoS One. 2015 Apr 13;10(4):e0122856. doi: 10.1371/journal.pone.0122856 (PMC4395337; doi:10.1371/journal.pone.0122856)

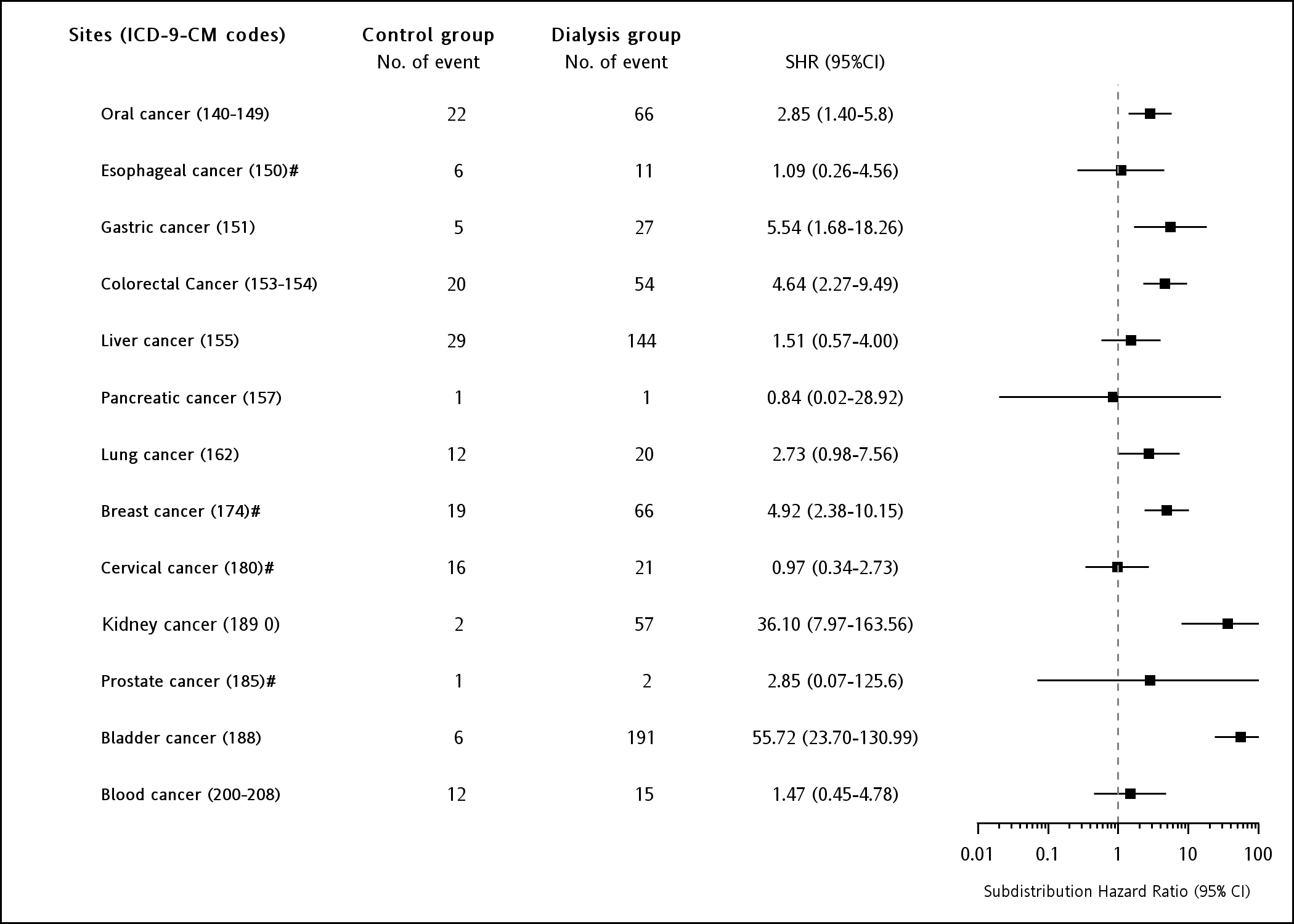

Supplement: S1 Fig — (TIF) [file pone.0122856.s001.tif]

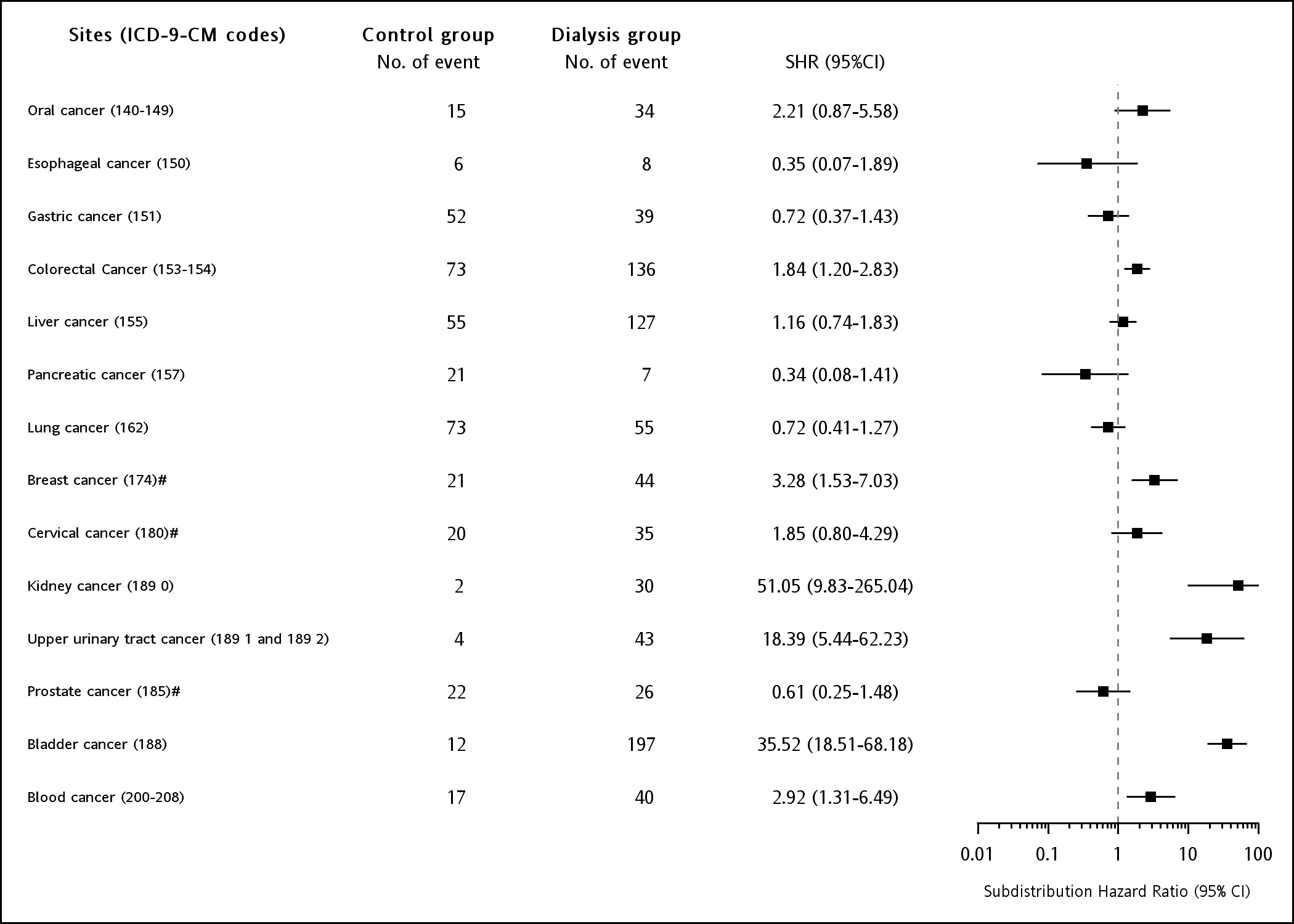

Supplement: S2 Fig — (TIF) [file pone.0122856.s002.tif]

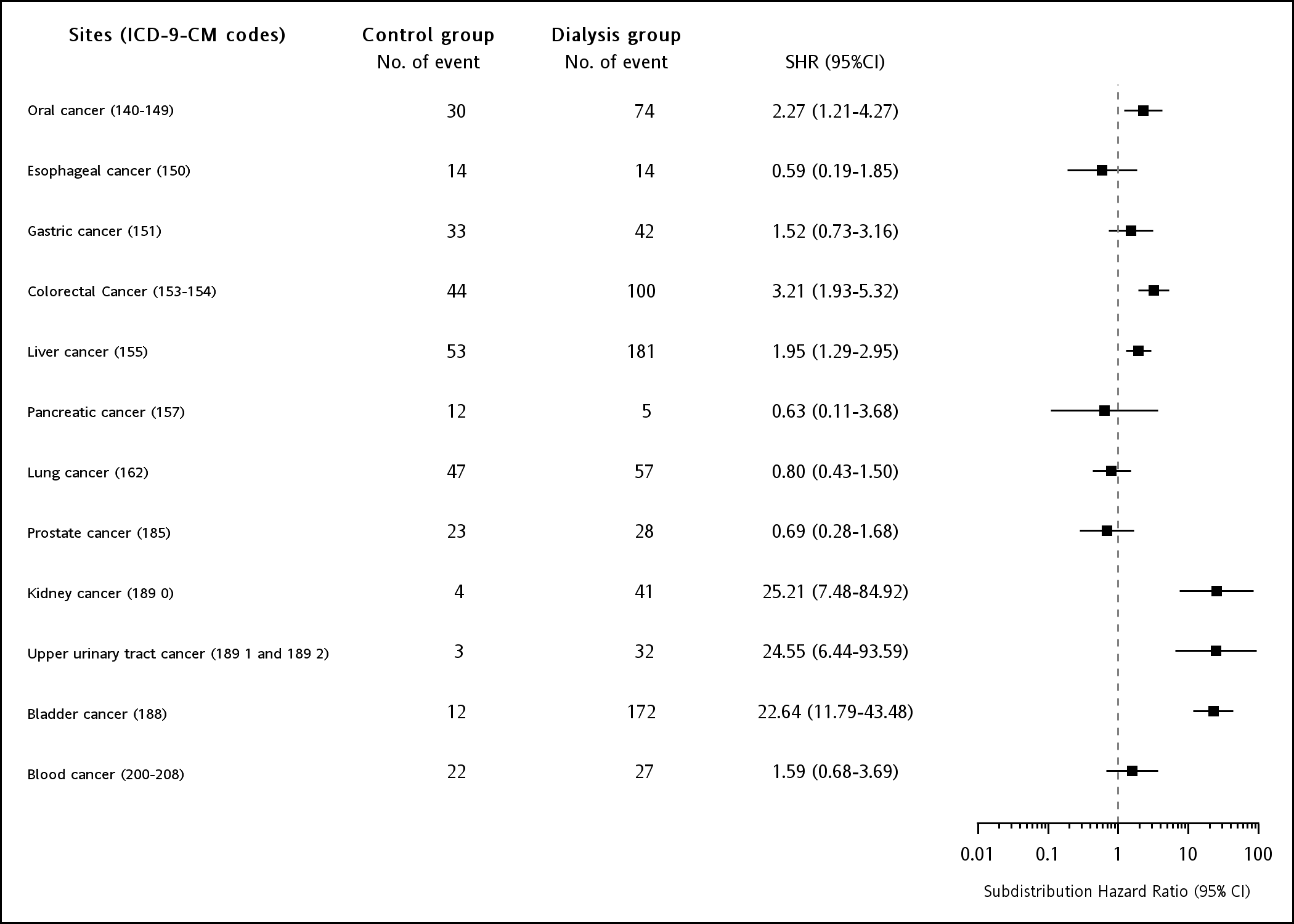

Supplement: S3 Fig — (TIF) [file pone.0122856.s003.tif]

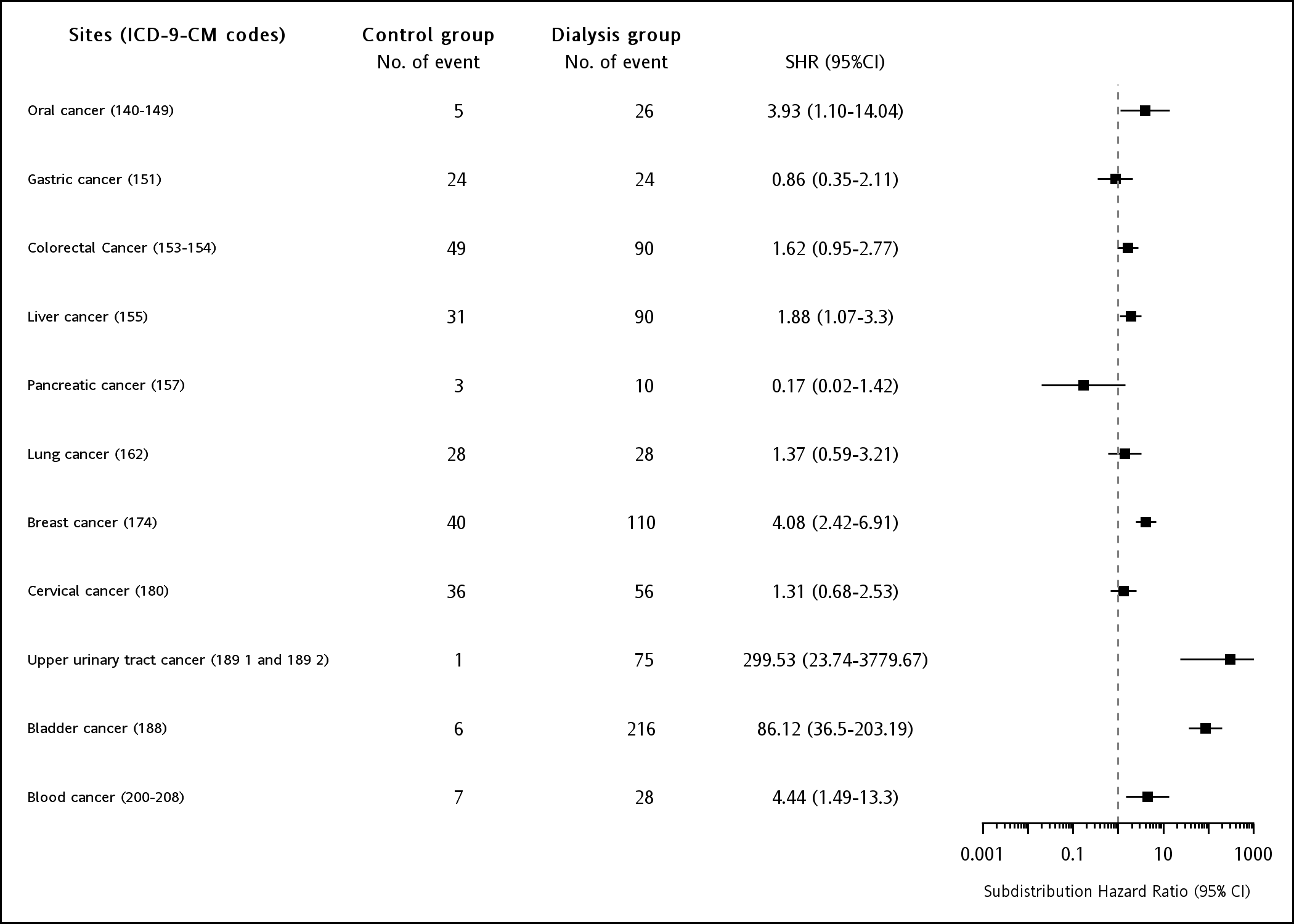

Supplement: S4 Fig — (TIF) [file pone.0122856.s004.tif]
