# Supplementary material for: Association of Dialysis with the Risks of Cancers
Source: PLoS One. 2015 Apr 13;10(4):e0122856. doi: 10.1371/journal.pone.0122856 (PMC4395337; doi:10.1371/journal.pone.0122856)
Supplement: S1 Table — (DOCX) [file pone.0122856.s005.docx]

Table S1. The corresponding ICD-9-CM codes for the diagnosis of disease in the study

| Disease | Corresponding ICD-9-CM codes |
| --- | --- |
| Acute coronary syndrome | 410, 411, 412 |
| Diabetes | 250 |
| Hypertension | 401-405 |
| Hyperlipidemia | 272 |
| [Chronic obstructive pulmonary disease](http://en.wikipedia.org/wiki/Chronic_obstructive_pulmonary_disease) | 491, 492, 496 |
| Cerebrovascular disease | 430-438 |
| Identified patients form the Registry for Catastrophic Illness Patient Database | |
| Malignant neoplasm | 140-208 |
| Chronic renal failure under regular dialysis: | |
| End-stage renal disease | 585 |
| Hypertensive heart or renal disease with renal failure | 403.01, 403.11,  403.91, 404.02,  404.03, 404.12,  404.13, 404.92, 404.93 |

Footnote: ICD-9-CM, International Classification of Disease, 9^th^ Revision, Clinical Modification
