# Supplementary material for: Association of Dialysis with the Risks of Cancers
Source: PLoS One. 2015 Apr 13;10(4):e0122856. doi: 10.1371/journal.pone.0122856 (PMC4395337; doi:10.1371/journal.pone.0122856)
Supplement: S2 Table — (DOCX) [file pone.0122856.s006.docx]

Table S2. The payment codes for the clinical treatment providing by Taiwan National Health Insurance

| Clinical treatment | Corresponding payment codes |
| --- | --- |
| Hemodialysis treatment per patient visit | 58001C, 58019C, 58020C, 58021C, 58022C, 58023C, 58024C, 58025C, 58027C, and 58029C. |
| Caring peritoneal dialysis per patient visit | 58002C, 58009A, 58009B, 58010A, 58010B, 58011A, 58011AB, 58011B, 58011C, 58012A, 58012B, 58017A, 58017B, 58017C, 58026C, 58028C. |
| Radical nephrectomy | 76007B, 76008B, 76027B, 76028B |
| Laparoscopic nephrectomy | 76021B |
| Partial nephrectomy | 7605B, 76031B |

Information was accessed at <http://www.nhi.gov.tw/Query/query4.aspx?menu=20&menu_id=712&WD_ID=832>
