# Supplementary material for: Association of Dialysis with the Risks of Cancers
Source: PLoS One. 2015 Apr 13;10(4):e0122856. doi: 10.1371/journal.pone.0122856 (PMC4395337; doi:10.1371/journal.pone.0122856)
Supplement: S3 Table — (DOCX) [file pone.0122856.s007.docx]

| Table S3. Diagnosed age and proportion of female in subjects with occurring cancer | | | |
| --- | --- | --- | --- |
| Age | Dialysis Group | Control Group | P-value |
| All cancer | 62.4±11.7 | 67.4±15.4 | <.001 |
| Oral cancer | 58.0±10.2 | 60.2±11.0 | .27 |
| Esophageal cancer | 62.4±11.8 | 63.6±9.5 | .76 |
| Gastric cancer | 63.6±11.2 | 72.6±10.5 | <.001 |
| Colorectal Cancer | 67.9±10.2 | 69.1±10.5 | .35 |
| Liver cancer | 60.0±11.4 | 65.1±9.6 | <.001 |
| Pancreatic cancer | 71.2±12.6 | 73.4±7.9 | .57 |
| Lung cancer | 68.7±9.0 | 70.4±8.9 | .23 |
| Hematologic cancer | 65.1±12.4 | 63.8±11.2 | .63 |
| Breast cancer | 57.9±13.5 | 60.6±10.6 | .26 |
| Cervical cancer | 62.8±10.2 | 65.0±13.5 | .38 |
| Prostate cancer | 71.6±6.6 | 75.7±8.9 | .06 |
| Kidney cancer | 58.0±10.8 | 59.1±10.8 | .85 |
| Upper urinary tract cancer | 60.1±11.0 | 72.3±7.4 | .03 |
| Bladder cancer | 62.9±11.2 | 68.1±12.7 | .06 |
|  |  |  |  |
| Female sex, % |  |  |  |
| All cancer | 52.4 | 45.0 | .001 |
| Oral cancer | 26.0 | 14.3 | .16 |
| Gastric cancer | 36.4 | 42.1 | .52 |
| Colorectal Cancer | 47.4 | 52.7 | .40 |
| Liver cancer | 33.2 | 36.9 | .53 |
| Pancreatic cancer | 37.5 | 45.5 | .70 |
| Lung cancer | 37.3 | 32.9 | .56 |
| Blood cancer | 50.9 | 24.1 | .02 |
| Kidney cancer | 52.9 | 0 | - |
| Upper urinary tract cancer | 70.1 | 25.0 | .06 |
| Bladder cancer | 55.7 | 33.3 | .06 |
| Data are expressed as percentages. Chi-Square test and independent t test is used to test the differences between the dialysis group and the control group. Statistical significance is defined as p value less than 0.05. | | | |
