# Supplementary material for: Association of Dialysis with the Risks of Cancers
Source: PLoS One. 2015 Apr 13;10(4):e0122856. doi: 10.1371/journal.pone.0122856 (PMC4395337; doi:10.1371/journal.pone.0122856)
Supplement: S4 Table — (DOCX) [file pone.0122856.s008.docx]

| Table S4. Diagnosed age by sex in subjects with occurring cancer | | | |
| --- | --- | --- | --- |
| Age | Dialysis group | Control group | P-value |
| Male | Mean±Sd (n) | Mean±Sd (n) |  |
| All cancer | 62.95±11.34 (886) | 68.13±10.47 (354) | <.001 |
| Oral cancer | 57.41±9.03 (74) | 61.68±10.75 (30) | .04 |
| Esophageal cancer | 61.32±11.47 (14) | 63.62±9.52 (14) | .57 |
| Gastric cancer | 64.87±11.06 (42) | 72.21±10.64 (33) | .005 |
| Colorectal Cancer | 66.91±10.15 (100) | 68.40±10.02 (44) | .42 |
| Liver cancer | 58.42±11.30 (181) | 65.18±9.89 (53) | <.001 |
| Pancreatic cancer | 69.85±12.41 (5) | 72.79±7.70 (12) | .56 |
| Lung cancer | 69.00±9.30 (57) | 71.06±8.92 (47) | .25 |
| Blood cancer | 66.60±11.03 (27) | 65.61±10.53 (22) | .75 |
| Prostate cancer | 71.56±6.58 (28) | 75.70±8.87 (23) | .06 |
| Kidney cancer | 58.54±10.26 (41) | 59.06±10.86 (4) | .92 |
| Upper urinary tract cancer | 61.57±10.23 (32) | 74.05±7.95 (3) | .05 |
| Bladder cancer | 63.89±11.72 (172) | 65.25±9.47 (12) | .70 |
|  |  |  |  |
| Female |  |  |  |
| All cancer | 61.94±11.92 (997) | 66.58±11.89 (290) | .001 |
| Oral cancer | 59.54±13.19 (26) | 51.58±8.78 (5) | .21 |
| Gastric cancer | 61.42±11.29 (24) | 73.05±10.52 (24) | <.001 |
| Colorectal Cancer | 68.94±10.20 (49) | 69.71±10.97 (90) | .68 |
| Liver cancer | 63.26±11.12 (31) | 64.99±9.10 (90) | .44 |
| Pancreatic cancer | 73.51±15.21 (10) | 74.23±8.57 (3) | .92 |
| Lung cancer | 68.09±8.79 (28) | 69.02±8.77 (28) | .69 |
| Blood cancer | 63.71±13.64 (28) | 58.14±12.06 (7) | .33 |
| Breast cancer | 57.94±13.46 (110) | 60.62±10.57 (40) | .26 |
| Cervical cancer | 62.79±10.25 (56) | 65.00±13.50 (36) | .38 |
| Kidney cancer | 57.6±11.4 (46) | - | - |
| Upper urinary tract cancer | 59.5±11.3 (75) | 66.9 (1) | - |
| Bladder cancer | 62.0±10.73 (216) | 73.7±17.06 (6) | .01 |
| Data are expressed as percentages. Chi-Square test and independent t test is used to test the differences between the dialysis group and the control group. Statistical significance is defined as p value less than 0.05. | | | |
